# Video Summarization for Sign Languages Using the Median of Entropy of Mean Frames Method

**DOI:** 10.3390/e20100748

**Published:** 2018-09-29

**Authors:** Shazia Saqib, Syed Asad Raza Kazmi

**Affiliations:** Department of Computer Science, Government College University, Lahore 54000, Pakistan

**Keywords:** entropy, keyframes, Shannon’s entropy, sign languages, video summarization, video skimming

## Abstract

Multimedia information requires large repositories of audio-video data. Retrieval and delivery of video content is a very time-consuming process and is a great challenge for researchers. An efficient approach for faster browsing of large video collections and more efficient content indexing and access is video summarization. Compression of data through extraction of keyframes is a solution to these challenges. A keyframe is a representative frame of the salient features of the video. The output frames must represent the original video in temporal order. The proposed research presents a method of keyframe extraction using the mean of consecutive *k* frames of video data. A sliding window of size k/2 is employed to select the frame that matches the median entropy value of the sliding window. This is called the Median of Entropy of Mean Frames (MME) method. MME is mean-based keyframes selection using the median of the entropy of the sliding window. The method was tested for more than 500 videos of sign language gestures and showed satisfactory results.

## 1. Introduction

**Figure 1:**
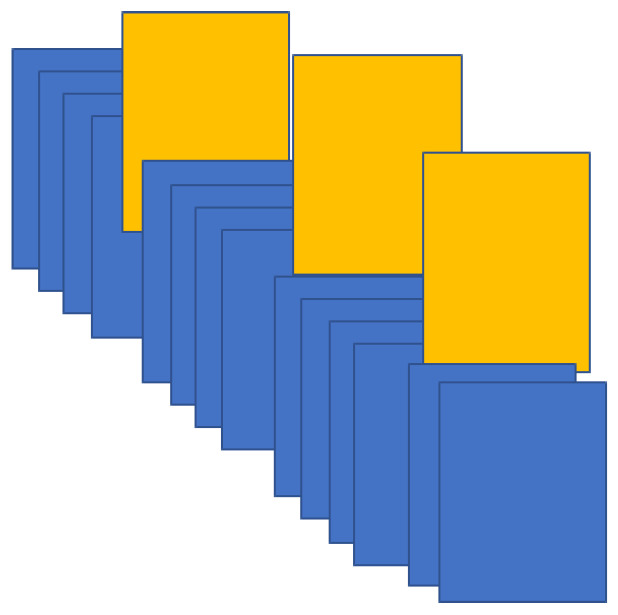
Video converted to frames and mean of *k* frames.

Gesture recognition is a giant leap toward the touch-free interface. The information conveyed through gestures is either in the form of static gestures or in the form of continuous gestures [1]. The continuous gestures are represented by videos [2]. A video itself cannot be recognized. A video needs to be summarized for analysis of its content. Video summarization is used to prepare a reduced size of the video in the form of frames that can be used for indexing or content analysis. This research aims at a keyframe extraction technique that can, in turn, be used for object recognition and information retrieval. Every video can be converted into frames. A keyframe refers to the image frame that represents the maximum information contained in a group of frames [3]. The keyframe defines the starting and ending points of any transition. The position of the keyframe tells us about the timing of any event. Combining all keyframes results in the abstract of the particular video. The idea of keyframe usage is very powerful as it saves a great deal of processing time and requires less storage. Figure 1 shows a few frames at a time; orange frames are the frames with mean values. Keyframes are basically the representative frames of a video. Using an appropriate technique, keyframes can be located among all frames of the video. These frames represent the video content and thus reduce the amount of storage and processing needed.

**Figure 2:**
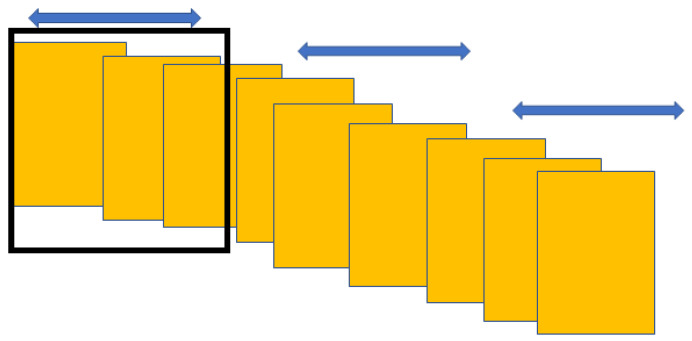
Keyframe selection using entropy measure through the sliding window of size k/2.

The selection of ”correct” keyframe is based on the application as well as the personal “definition” of what the summary should represent. Figure 2 shows the mean frames in a sliding window whose median of entropy is being calculated. The size of the sliding window is chosen such that it has an odd number of elements.

Researchers have described keyframe extraction into either “sequence-based approaches” or “cluster-based approaches” [4]. The first type of approaches uses the temporal information and visual features to identify the keyframes. Consecutive frames are compared and the variation in consecutive frames is estimated. When a substantial change in the frame is detected, that frame is selected as the keyframe. Cluster-based approaches divide the video stream into shots. The frames that represent the shot are chosen as candidate keyframes. The clustering process should maintain the temporal order of the frames [4].

The process of selecting keyframes passes through video information analysis, meaningful clip selection, and output generation. For a good summary of video information, we must determine salient features, the descriptors in the visual component, the audio component if any, and the textual components such as closed captions. A shot can change by a “CUT”, which is a sudden change between two adjacent frames, or a “FADE”, which occurs by a steady change in brightness. Another is “DISSOLVE”, which is similar to FADE but is sandwiched between two shots. One scene gets dimmer and the incoming scene gets brighter, and the 2nd shot finally replaces the first one [4]. All the methods of video summarization are grouped into the following:

### 1.1. Static Video Summarization

The video is sampled either uniformly or randomly. The complete video is divided into frames. Out of these frames, one or more will be representative of the content of the video, helping in generating video summaries [4].

### 1.2. Methods Based on Clustering Techniques

These techniques combine similar frames/shots. Some features are then extracted from this group of frames. Based on this, one or more frames are extracted from the cluster. Different features such as luminance, color histogram, a motion vector, and *k*-means clustering are used in making the decision for keyframe selection [4].

### 1.3. Dynamic Video Summarization

This is also called video skimming, which is actually a summary video of all the important scenes from an input stream. It forms an abstraction of the video. Singular Value Decomposition (SVD), and motion model and semantic analysis, are the few techniques that are used for dynamic video summarization [4].

The rest of the paper is organized as follows: Section 2 covers related work, Section 3 shows the algorithm and experimental work. Section 4 elaborates the results of the experiment, and Section 5 concludes and suggests future work.

## 2. Related Work

A great deal of work has been done on video summarization. Sheena and Narayanan used the histogram of consecutive frames. In this method, the threshold difference of histograms is calculated to find keyframe from video data from the KTH action database. Their algorithm is good both in terms of fidelity value as well as compression ratio [3]. Khattabi et al. analyzed the static and dynamic methods of producing video summaries [4]. Tsai et al. have related transmitted information and image noise, they investigated the effect of noise on blurring. They further analyzed the use of smoothing filters for improving the noise and blurring, their results gave reasonable performance in medical imaging [5]. Fauvet et al. used the computation of the dominant image motion and the geometrical properties that result in a change in a frame in the considered shot. They improved their own technique at computational cost using an energy function. They tested their technique on sports videos and obtained satisfactory results [6]. Vasconcelos et al. presented a technique for characterization and analysis of video data. They used Bayesian architecture to analyze the content of videos on a semantic basis [7]. Mikolajczyk et al. compared the detection rate with the false positive rate. They used differential invariants, steerable filters, Scale Invariant Feature Transform (SIFT) descriptors, moment invariants, complex filters, and cross-correlation. Their research shows that SIFT descriptors yielded the best results. Steerable filters also proved to be a good choice [8]. Sebastian et al. proposed a technique that divides the frames of the video into blocks. They used the mean, variance, skew, and kurtosis histogram of every block and compared them with the corresponding blocks of the next frame. They selected the frame with the highest mean as the keyframe. The method is based on the color distribution [9]. Supriya Kamoji et al. captured the motion in a video to find the keyframes. To analyze this motion, block matching techniques based on Diamond Search and Three Step Search were compared. The comparison process is on the varied nature of videos. The summarization factor was increased at the cost of precision during the summarization process [10]. Mentzelopoulos et al. compared all of the current keyframe extraction algorithms. They proposed the use of Entropy-Difference for spatial frame segmentation [11].

Cahuina et al. proposed a technique based on local descriptors for semantic video summarization and tested the technique on 100 videos. Their technique achieved a recognition level of 99%. They used color information with local descriptors to produce video summaries [12]. Shi et al. proposed a key frame extraction method for video copyright protection. Their technique is based on the difference of frames using features such as color and structure. For final results, optimization is done on a number of keyframes that have been selected [13]. Zhao et al. proposed the use of local motion features extracted from their neighborhood. Their method uses a hierarchical spatial pyramid structure giving very good results over standard benchmark datasets [14]. Hasebe et al. proposed a new method to find the keyframes for input videos. The technique works in the wavelet transform domain. As a first step, shot boundaries are sorted out so that initial keyframes may be defined. Secondly, feature vectors are grouped into clusters for these selected frames. The results are tested on the basis of processing speed and precision rates [15]. Mahmoud et al. have suggested the use of VGRAPH that uses color as well as texture features. The video is divided into shots based on color features. The technique uses a nearest neighbor graph using textural features [16].

Ciocca et al. proposed an algorithm based on the difference between two consecutive frames of a video sequence and used the visual content changes. They used a color histogram, wavelet statistics, and an edge direction histogram. Similarity measures are determined and combined with the frame difference. The method even detects very minor changes. The proposed method dynamically selects a variable number of keyframes from different shots [17]. Ejaz et al. combined the features of Red Green Blue (RGB) color channels, histograms, and moments to find the keyframes. The technique is adaptive as it combines current and old iterations. The summaries produced by these techniques are as good as those created by humans [18]. Rajendra et al. reviewed previous work on content-based information processing for multimedia data. They focused on how to browse andhow to add new features, learning, effective computing semantic queries, high-performance indexing, and evaluation techniques [19]. Girgensohn et al. designed an algorithm to find keyframes that represent the input video. This technique can determine keyframes from a video by clustering frames. Each cluster has a representative frame, and some clusters are not considered and left unprocessed on temporal grounds [20]. Guan et al. suggested a keypoint-based framework for selecting keyframes using local features. The resultant frames represent video without any redundancy [21]. Asade et al. suggested an algorithm to extract static video summaries. Their technique is based on fuzzy c-means clustering. The frame with the highest membership grade for any cluster is selected as a keyframe. Their method gives a lower error rate with a higher accuracy level [22].

Zhang et al. used the similarity distance of the adjacent frames to adjust the threshold input adaptive algorithm. They then used the Iterative Self-Organizing Data Analysis Technique (ISODATA) to cluster frames into classes automatically. Their algorithm focuses on different motion types reliably and efficiently. Their results were tested using metrics that analyzed for the reconstructed motion and the mean absolute error value [23]. Dong et al. suggested an algorithm for keyframe selection and recognition method for robust markerless real-time camera tracking. Their technique used one offline and one online module—offline uses a number of images and online uses a video to detect a pose. Their technique reduces redundancy and, at the same time, produces a best possible set of frames [24]. Kim et al. proposed a technique that generates panoramic images from web-based geographic information systems. Their algorithm performs data fusion, crowd sourcing, and recent advances in media processing. Their work shows that a great deal of time can be saved if “geospatial metadata” is used without any compromise on image quality [25].

Mei et al. generated audio streams, compressed images, and metadata for motion information and temporal structure. Their technique works at a very low compression rate. The proposed Near-Lossless Semantic Video Summarization (NLSS) method is effectively used for visualization, indexing, browsing, duplicate detection, concept detection, etc. The NLSS is tested on TREC Video Retrieval Evaluation (TRECVID) and other video collections, showing that it significantly reduces storage consumption while giving high-level semantic fidelity [26]. Shroung et al. used the image difference and classification theory to identify keyframes from video captured using ordinary mobile or laptop cameras, yielding a highly accurate video summary. These video frames are used for dynamic sign recognition [27]. Vázquez-Martín et al. utilized consecutive frames and their features. They built a graph using these features and used clustering to partition the graph [28]. Khurana et al. used the edge detection and the difference of this value between the consecutive frames. The frames matching a threshold are treated as keyframes [29]. Thakre et al. proposed a technique for keyframe selection of compressed video shots using the adaptive threshold method working on 200 plus video clips [30].

Wang et al. elaborated the important issues in information theory and discussed the use of these concepts in visualization in relating data communication to data visualization [31]. Entropy has been used for image segmentation by [32,33,34,35], covering various types of available entropy algorithms. Sabuncu discussed the use of different entropic measures that can be used for image registration [36]. Ratsamee et al. proposed finding a keyframe that is based on image quality measurements such as color, sharpness, noise, etc. However, a biosensor is required to determine human excitement [37]. Angadi et al. proposed a technique that uses a fuzzy c-means clustering algorithm. The technique merges keyframes in a timewise order [38]. Yuan et al. used a Deep Side Semantic Embedding (DSSE) model to select keyframes. They correlated two uni-modal autoencoders, yielding side information and video frames. They tested their work on the Title-based Video Summarization (TVSum50) dataset [39]. Chen et al. employed the visual and textual features of videos. Their technique uses their previously reviewed frames and posted comments [40]. Panda et al. used video-level annotation for summarizing web videos. They used Deep Convolutional Neural Network (3D CNN) architecture for video-level annotation [41]. Mahasseni et al. used a deep summarizer network that used a summarizer autoencoder named a Long Short-term Memory Network (LSTM) [42]. Jeoung et al. proposed a technique for a static summary of consumer videos. They completed the process in two steps: first they skimmed the video and then performed content-aware clustering with keyframe selection [43]. Yoon et al. proposed an approach based on learning principal person appearance [44].

De Avila et al. proposed Video SUMMarization (VSUMM) for producing static video summaries. The method is based on color feature extraction from video frames and a k-means clustering algorithm. The work was compared with manually created static summaries, demonstrating the high accuracy of the proposed VSUMM technique. The technique improves on visual features, their fusion, and the estimation of the number of clusters [45]. Kanehira et al. proposed Fisher’s discriminant criteria for inner-summary, inner-group, and between-group variances defined on the feature representation of summary [46]. Manis et al. have proposed the Bubble Entropy to rank the elements inside the vectors for doing reallocation to sort these elements [47]. Athitsos et al. have designed the dataset ASL Lexicon Video Dataset to develop a computer vision system that helps in recognizing the meaning of an ASL sign. The dataset can be a benchmark for a variety of computer vision and machine learning methods [48]. PUN proposed a technique for threshold selection method to segment images using the entropy of the grey level histogram dividing them into two-level images [49]. Sluder and David have proposed to use averaging to reduce noise in an image. The magnitude of noise drops by the square root of the number of images averaged [50]. Panagiotakis et al. suggested using three iso-content principles (Iso-Content Error, Iso-Content Distance, and Iso-Content Distortion) so that the selected keyframes are generated according to the algorithm used. The technique used both Supervised and Unsupervised approaches. The proposed technique requires an improvement in the temporal order of frames from different shots [51]. Song et al. presented Title-based Video Summarization (TVSum), which is an unsupervised video summarization framework that uses video labeling to summarize the video. The co-archetypal analysis is done for canonical patterns between two sets of data. However, they need to improve the image collection procedure and to make use of metadata to produce the video summary [52]. Mei et al. proposed video summarization based on a constrained Minimum Sparse Reconstruction (MSR) model by recreating a video using keyframes generated with minimum possible frames. A Percentage of Reconstruction (POR) criterion is used to determine the length of the summary. Their technique summarizes both structured videos and the consumer videos [53]. Ajmal et al. used the Histogram of Oriented Gradient (HOG) using a Support Vector Machine (SVM) classifier. The Kalman filter in the algorithm determines the track of each person [54].

## 3. The Proposed Median of Entropy of Mean Frames (MME) Technique for Keyframe Selection

The proposed technique uses the concept of the mean and then applies the median of the entropy to the resultant images for video summarization. The resultant keyframes thus generated will be used for continuous gesture recognition. The technique uses the mean of *k* images. It then takes a group of k/2 mean frames at a time and determines their entropy. The median of the entropy measure is calculated to select the keyframes. The value of *k* is chosen such that k/2 is odd, for easy selection of keyframes.

### 3.1. Mean

The mean is a very important measure in digital image processing. It is used in spatial filtering and is helpful in noise reduction. The mean of *k* frames is defined as

(1) $${\hat{f}}_{l}\left(i,j\right)=\frac{\sum_{m=1}^{k}\sum_{i=1}^{n}\sum_{j=1}^{n}f_{m}\left(i,j\right)}{k}.$$

Here, ${\hat{f}}_{l}\left(i,j\right)$ shows the lth mean of *k* images. $\sum_{m=1}^{k}\sum_{i=1}^{n}\sum_{j=1}^{n}f_{m}\left(i,j\right)$ is the sum of *k* frames. $\sum_{i=1}^{n}\sum_{j=1}^{n}f_{m}\left(i,j\right)$ shows the *m*th frame.

### 3.2. Entropy

Entropy is the measure of randomness (or uncertainty) in an image. It is a measure of the information transmitted [5]. The concept was given by Claude Shannon and is called Shannon’s entropy [35]. Maximum entropy, Renvi entropy, Tsallis entropy, spatial entropy, minimum entropy, conditional entropy, cross-entropy, relative entropy, and fuzzy entropy are used for image segmentation, image registration, image compression, image reconstruction, and edge detection in gray level images [33]. Bubble entropy investigates the rank of the members of the collection of data and determines a method of sorting these elements. Bubble entropy is considered a good option in biomedical signal analysis and interpretation [47].

Entropy is a measure of the spread of states in which a system can adjust. A system with low entropy will have a small number of such states, while a high entropy system will be spread over a large number of states. Suppose X is a random variable consisting of following $X_{1},X_{2}\ldots ,X_{l}$. The variable *X* has a probability distribution $p\left(x\right)=\left(p_{1},p_{2},p_{3}\ldots p_{l}\right)$, which is used for the calculation of the Shannon’s entropy. The entropy of an l-state system is given as

(2) $$H=-\sum_{k=0}^{l-1}p_{k}\log_{b}p_{k}$$

where $p_{i}$ is the probability of occurrence of the event *i* and $\sum_{i=0}^{l-1}p_{i}=1$. b is the base of the algorithm and is usually 2. If $P(x_{i})=0$ for some *i*, then the multiplier $0log_{b}0$ is considered as zero, which is consistent with the limit [36]. The term $\log(\frac{1}{p_{i}})$ shows the uncertainty associated with the corresponding outcome or can also be viewed as the amount of information gained by observing that outcome. Entropy represents the statistical average of uncertainty or information. The number of pixels in the image is *n*, while $n_{k}$ represents a total number of pixels at level *k*.

(3) $$P_{k}=\frac{n_{k}}{n}$$

where *l* is the total number of gray levels, and $P_{k}$ is the probability associated with each gray level *k*. The value of entropy is highest when samples are equally likely and when H(p1…pl)≤log(l) [49].

The video summarization process involves the following stages:

- **Input Video**. This is the video that is to be converted into keyframes. It can be in any standard format.
- **Frame Extraction**. Every video is basically a sequence of a finite number of still images called frames. These frames occupy a large amount of memory. The frame rate is about 20–30 frames per second (FPS). Movies are shown at a rate of almost 24 fps. In some countries, it is 25 fps. In North America and Japan, the movies are shown at 29.97 fps. In other image processing applications, it is usually at 30 frames per second. Other common frame rates are usually multiples of these [19]. It has been found that, usually, 1–2 frames per second creates the illusion of movement. The rest of the frames show almost the same scene repeatedly [30].
- **Feature Extraction**. This process can be based on features such as colors, edges, or motion features. Some algorithms use other low-level features such as color histograms, frame correlations, and edge histograms [19].

**Figure 3:**
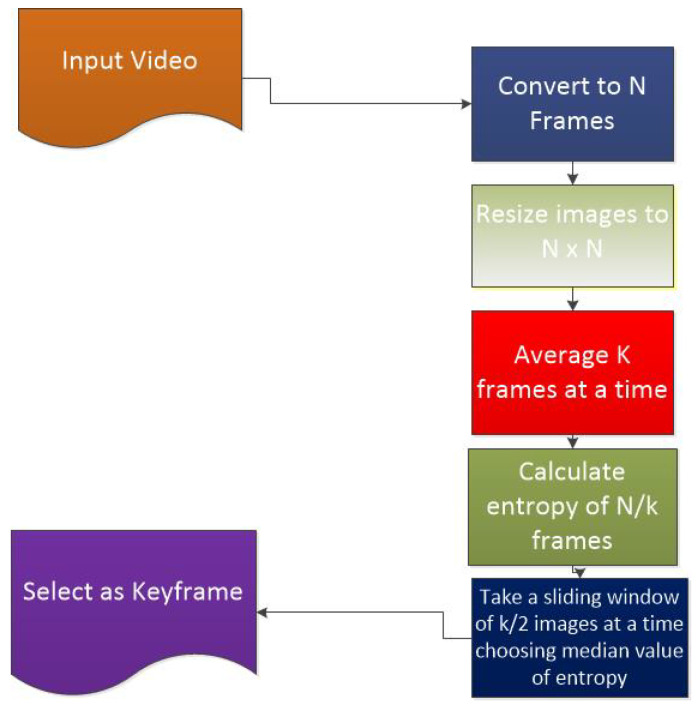
Flow of the procedure to extract keyframes.

Figure 3 elaborates the mechanism used in the proposed solution. It starts by capturing input video. The video is then converted to frames. Frames are then preprocessed and resized to an appropriate dimension. The proposed algorithm then takes the mean of k frames at a time, thus reducing images from *n* to *n*/*k*. After this, a sliding window of size *k*/2 is applied to the resultant frames, and in each window the frame with the median value of entropy is selected.

### 3.3. Algorithm to Find Keyframes

The technique uses the following **pseudocode**:

Input: The video.
Output: $kf_{1}$,$kf_{2}$, $kf_{3}$ …$kf_{tkfr}$, where tkfr represents total keyframes
procedure:
convert the video to frames $f_{1},f_{2},f_{3}\ldots f_{n}$;
resize each frame to an image size of n×n (in this proposed technique, we chose n=100);
initialize l to 1;
$\forall f_{i}\ldots 1\leq i\leq n$;
$\mu f_{l}=\frac{\sum_{i=1}^{k}f_{i}}{k}$;
increment l by 1;
reduce the frame count to n/k;
consider 1st sliding window of size k/2;
calculate entropy of frame *l*, l+1, l+2 …;
compare the frames in the sliding windows;
choose a frame with the median value of entropy;
slide window to the next k/2 consecutive frames.

**Algorithm 1:** Keyframe Extraction through the proposed MME method.

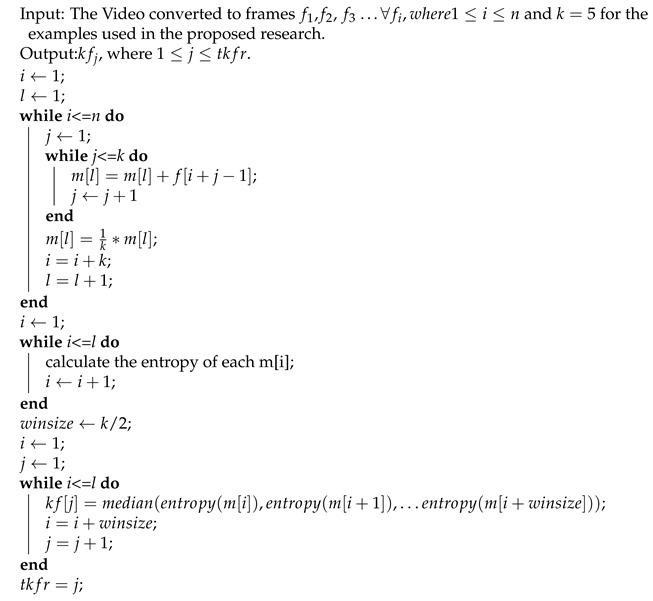



The proposed Algorithm 1 can be used for any type of video, but it has also been tested rigorously for continuous gestures. We tested this algorithm on several videos. The complexity of the algorithm is $O(n^{2})$. As a test case for the proposed technique, we took an example of a video of a gesture for the word **dress** in Pakistan Sign Language, which is 3 s long. It consists of almost 90 frames. In the first loop, five frames are averaged at a time, yielding 17 frames. We continued until all frames had been processed. Using computed entropy, we designed a sliding window of size 3 frames. Later on, the median of the entropies of the frames in the window was calculated. Using a 3 s video, we obtained six keyframes. The compression ratio (CR) is determined by

(4) CR=keyframes/totalframes.

A low CR represents an efficient technique. Fidelity is another measure to determine the efficiency of the keyframe selection algorithm. It is the maximum of the minimum of the distance between keyframes and the individual frames.

(5) $$d_{j}=min\{dis\left(kf_{j},f_{i}\right)\}.$$

(6) $$fidelity=max\{d_{j}\}.$$

Fidelity basically determines how effectively an algorithm maintains the global content of the original video [20].

## 4. Results and Analysis

**Figure 4:**
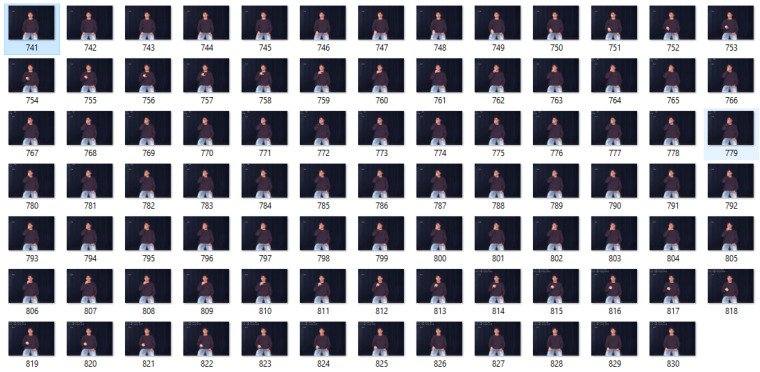
Frames in the video of a gesture for the word **bird** from American Sign Language.

The algorithm was tested on a number of videos, and a few examples are presented here. The technique was applied to the ASL LexiconVideo Dataset, containing thousands of distinct sign classes of American Sign Language [48]. Figure 4 shows extracted frames from a video of a gesture of the word **bird**, which is 3 s long.

**Figure 5:**
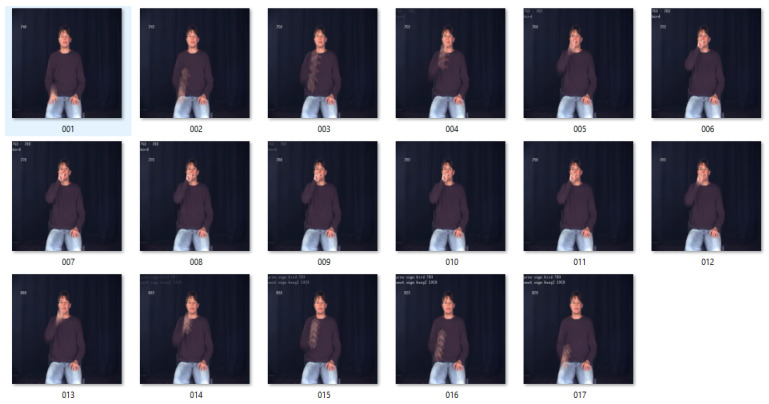
Average frames for the video of the gesture for the word **bird**.

Figure 5 shows the 17 mean frames calculated by taking the mean of *k* frames using the video of the gesture for the word **bird** for k=5.

**Figure 6:**
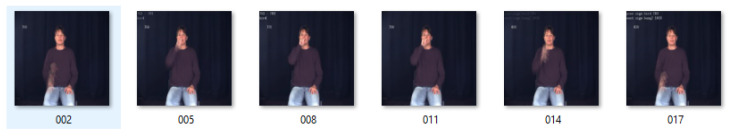
Keyframes generated by using the MME methodfor k=5.

Figure 6 shows the keyframes generated with the proposed MME technique using the median of the entropy of the mean frames using a sliding window of size k/2.

**Figure 7:**
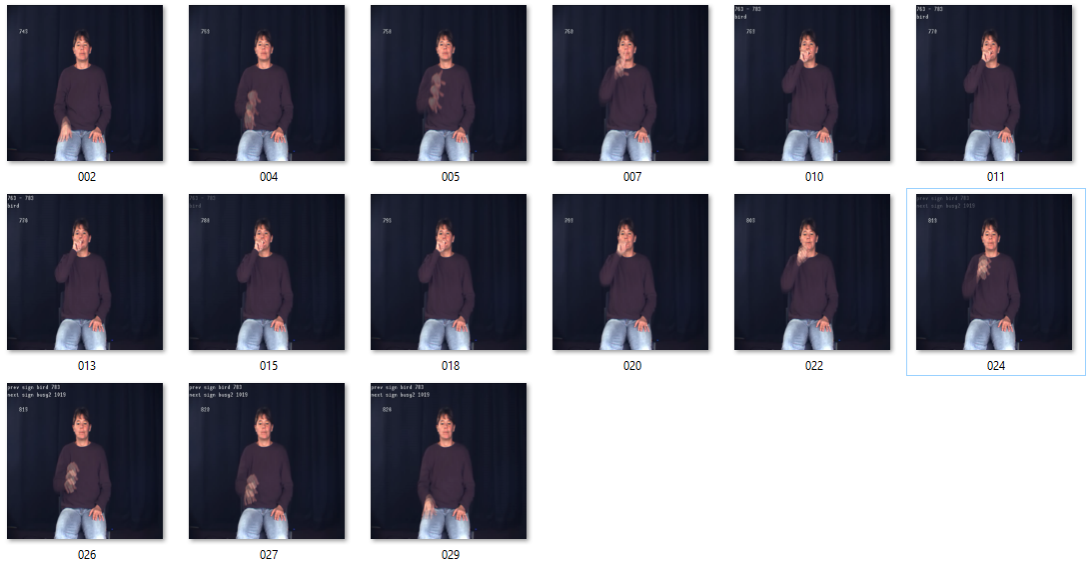
Keyframes generated by using the MME method for k=3.

Figure 7 shows the median of entropy from the mean frames using a sliding window of k/2 while k=3. The video of the gesture for the word **bird** changes frames at a faster pace. Therefore, for the faster videos, we decreased the value of the *k*; for the slower videos, we increased the value of *k* accordingly.

**Figure 8:**
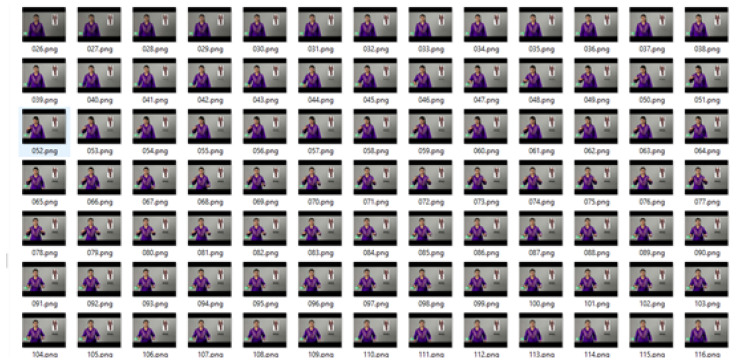
Frames in the video of a gesture for the word **dress** in Pakistan Sign Language.

In another scenario, the video of the gesture for the word **dress** was converted to frames. It was also 3 s long. It was converted to 90 frames, as shown below in Figure 8.

**Figure 9:**
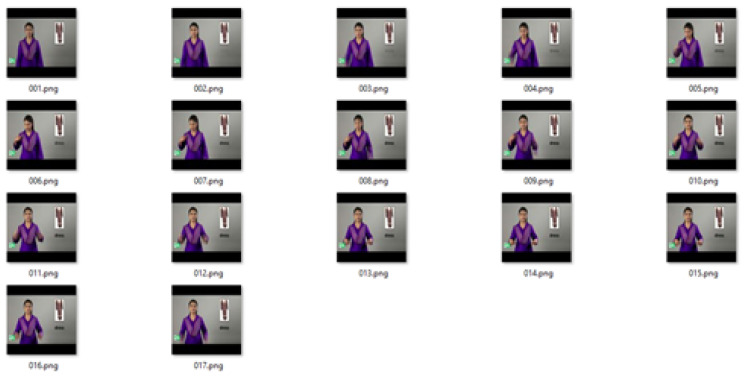
The mean of input frames of a video of the gestures representing the word **dress** in Pakistan Sign Language.

The mean for these frames was calculated taking 5 frames at a time, so we obtained 17 frames after the process was applied. Figure 9 represents the frames calculated using the mean of 5 frames at a time.

**Figure 10:**
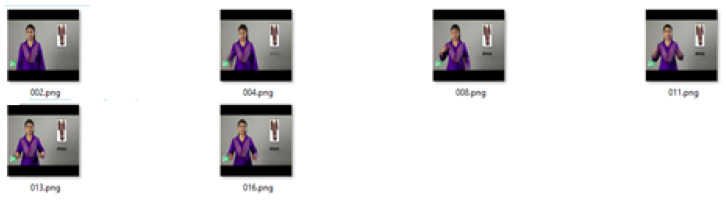
Keyframes generated by using the proposed MME technique.

The entropy was calculated for all frames after taking the mean. A sliding window was applied to three frames at a time. The entropy value of all images was calculated, and the frame representing the median of these frames was selected as the keyframe. Once a keyframe was selected, the sliding window moved to the next three frames. We obtained six frames as the keyframes. Figure 10 shows selected keyframes.

**Figure 11:**
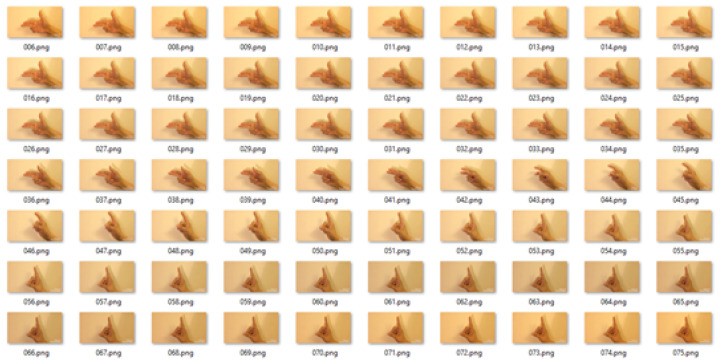
Frames extracted from the video of the gesture for the word **letter** in Pakistan Sign Language.

In another example, a video of the gesture for the word **letter** in Pakistan Sign Language was chosen to test the proposed algorithm. For a video 2 s long, we had approximately 70 frames. Figure 11 shows the frames extracted from the video of the gesture for the word **letter**.

**Figure 12:**
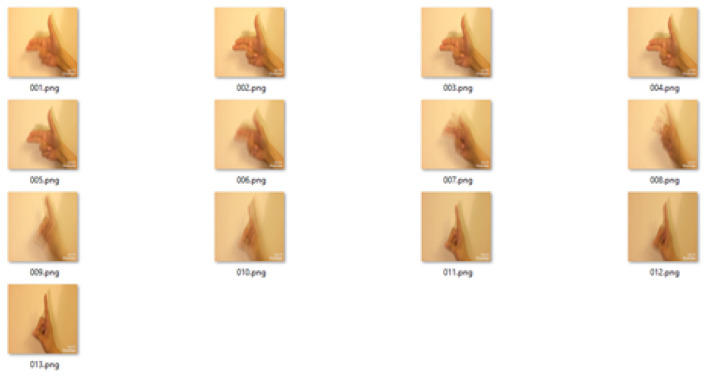
Average frames for the video of the gesture for the word **letter**.

We obtained 13 frames after applying a mean 5 frames at a time. Figure 12 shows the resultant frames after taking the mean of the input frames.

**Figure 13:**
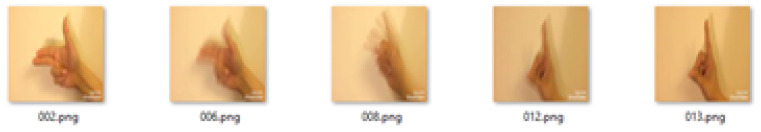
Keyframes for the word letter using the proposed MME technique.

In the last step, using the sliding window of size 3, we obtained five frames as keyframes. Figure 13 shows the keyframes after applying a median on the value of entropy.

Table 1 shows the result of the proposed technique in various videos of Pakistan Sign Language gestures. Its first column shows the input video, and the second column shows the total number of frames from the input video. The table also provides the video duration in seconds, the number of frames after applying the mean, the number of frames after applying the median value of entropy, and the percentage of compression ratio.

Table 2 shows the results of the proposed technique and the technique based on the simple mean and the threshold of entropy.

**Figure 14:**
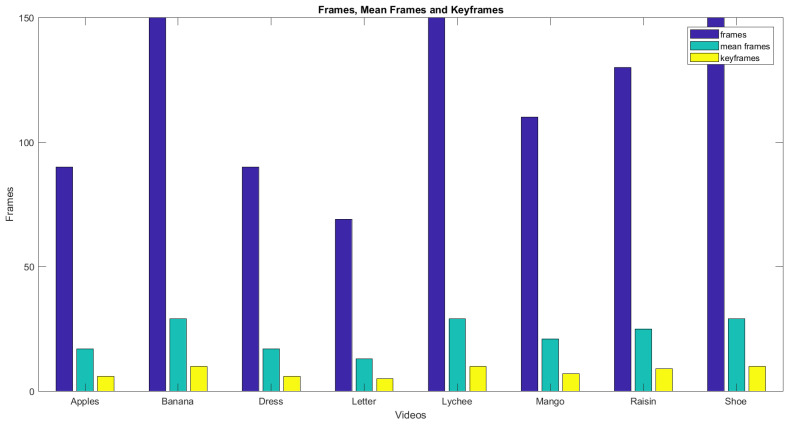
Frames, mean frames, and keyframes using the proposed MME.

**Figure 15:**
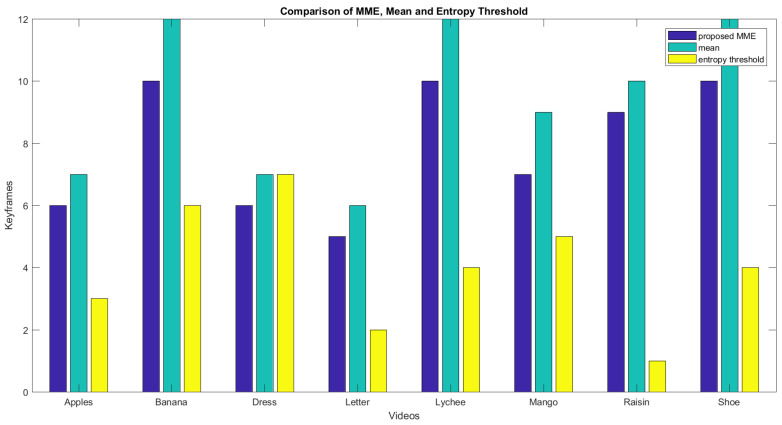
The amount of keyframes using the proposed MME, simple mean, and simple threshold of entropy methods.

Figure 14 shows the graph of the initial number of frames, the number of frames after the mean, and the number of frames after the sliding window operation. Blue bars show the total frames, and light blue bars show the number of frames after taking the mean. Yellow bars represent the resultant number of frames from the proposed MME technique. Figure 15 shows the keyframeextracted using the different techniques. The graph confirms that the proposed technique has an advantage over the techniques using simple mean or simple entropy threshold values. The simple mean can generate too many frames. Secondly, increasing the number of frames in calculating the mean beyond a certain limit is an expensive process, as it increases the required computational time. It grows with the number of images as well as the image size. The entropy threshold technique has its own weaknesses, as the selection of the threshold is a very challenging task. It may fail to deliver good results for certain videos. For the video of the dynamic gesture for the word **raisins**, a 4.5 s long video, the technique generated only one keyframe.

The results were also compared with other existing techniques. The proposed technique achieves accuracy comparable to those provided by [3,10,30,38,54]. It can be tested for qualitative as well as quantitative features, but an actual test is only valid if the video summary is used in the applications for which it was created. Table 3 shows the compression ratios of the proposed technique along with these other techniques. The proposed method performs fairly well in terms of this metric.

The proposed technique uses an average of *k* frames with a median of entropy using a sliding window of size k/2. The technique incorporates the advantage of taking the mean of consecutive frames. The noise is reduced at the rate of the square root of the number of frames that are used in taking the mean. Therefore, when we average n frames at a time, the noise in the images is reduced by $\sqrt{n}$ [50]. This implies that averaging 5 frames reduces noise by a factor of 2. However, it is a time-consuming process to take an average of frames depending on the capability of the device, as 1/30 of a second is required to average each frame. The technique based on simple averaging loses sharp transitions, and the selected keyframes might not produce an appropriate video summary. With simple entropy, selection of the threshold value is a difficult task. For faster videos such as those provided in the ASL LexiconVideo Dataset or videos of rapidly moving objects, we can select lower values of k for better results as shown in Figure 7.

## 5. Conclusions

Keyframe selection is an active area of research and plays a pivotal role in video summarization. A keyframe is one of the most efficient methods of obtaining a summary of the video content. The temporal order of the frames is maintained by the proposed MME technique. The frame average count k and sliding windows size k/2 can be changed depending on the nature of the video. For dynamic gestures, the value of *k* in the range 5–15 is preferential. Selecting a reasonable size of *k* rules out the possibility of missing frames in the proposed MME technique. Another distinct quality of the proposed technique is the distance of keyframes. We obtained keyframes that were at most k×k/2 frames apart. A few redundant frames may have been added, but thereis a much lower chance of losing important information. The system is designed to provide input to video recognition in the form of images that tell the story of the input video by different signers. Adjusting the value of k accordingly or adding a mechanism to learn the value of k makes the system workable, even for very fast or very slow videos. The effectiveness of the MME method is, however, compromised for very rapid changes in a scene. In the future, methods with improved criteria in terms of the mean rate and the sliding window size will be designed. Integrating appropriate filters to remove noise from the final selected keyframes may improve the proposed technique.

## Figures and Tables

**Table 1:** Videos converted to frames using MME.

Query Video	Frames	Video Duration	Frames after Taking Mean	Keyframes Extracted	Compression Ratio (%)
dress	90	3	17	6	6.67
letter	69	2	13	5	7.24
Apple	90	3	17	6	6.67
Banana	150	5	29	10	6.66
Raisin	130	4.5	25	9	6.6
Lychee	150	5	29	10	6.66
Shoe	150	5	29	10	6.66
mango	110	3.5	21	7	6.36

**Table 2:** Comparison of the Proposed MME with Simple Mean and Simple Entropy.

Query Video	Frames	Keyframes Extracted by the Proposed MME	Keyframes Using Mean	Keyframes Using Threshold of Entropy
dress	90	6	7	7
Khatt (letter)	69	5	6	2
Apple	90	6	7	3
Banana	150	10	12	6
Raisin	130	9	10	1
Lychee	150	10	12	4
Shoe	150	10	12	4
mango	110	7	9	5

**Table 3:** Comparison of some existing techniques.

Technique Name	Compression Ratio (%)
Analysis of Histograms of Video Frames using Statistical Methods [3]	7.08
Video Summarization Using Motion Activity Descriptors [10]	4.25
Keyframe Extraction of Compressed Video Shots using Adaptive Threshold Method [30]	4.5
Entropy-Based Fuzzy C-Means Clustering and KeyFrame Extraction [38]	8.4
Video Summarization for Sign Languages using MME	6.7
Human Motion Trajectory Analysis Based Video Summarization [54]	6.74
